# Supplementary material for: A designer FG-Nup that reconstitutes the selective transport barrier of the nuclear pore complex
Source: Nat Commun. 2021 Mar 31;12:2010. doi: 10.1038/s41467-021-22293-y (PMC8012357; doi:10.1038/s41467-021-22293-y)
Supplement: Supplementary file 1 — Supplementary Information [file 41467_2021_22293_MOESM1_ESM.pdf]

Supplementary information

A designer FG-Nup that reconstitutes the selective transport barrier of the Nuclear Pore Complex

Alessio Fragasso[1*], Hendrik W. de Vries[2*], John Andersson[3], Eli van der Sluis[1], Erik van der Giessen[2], Andreas Dahlin[3], Patrick R. Onck[2$], Cees Dekker[1$]

[1]Department of Bionanoscience, Kavli Institute of Nanoscience, Delft University of Technology, Van der Maasweg 9, 2629 HZ Delft, The Netherlands.
[2]Zernike Institute for Advanced Materials, University of Groningen, Nijenborgh 4, 9747AG, Groningen, Netherlands.
[3]Department of Chemistry and Chemical Engineering, Chalmers University of Technology, Kemivägen 10, SE-412 96, Gothenburg, Sweden.

[*]These authors contributed equally.

[$]Corresponding authors: p.r.onck@rug.nl, c.dekker@tudelft.nl

Overview of the Supporting information:

Supplementary Note 1 – Amino acid sequences of all proteins used in this work (NupX, Nsp1, Nsp1-S, Kap95, BSA).
Supplementary Table 1 – Overview of measured Stokes diameters for the protein used by DLS.
Supplementary Table 2 – Sequences of 25 NupX variants.
Supplementary Table 3 – Simulation parameters used for MD-simulations.
Supplementary Table 4 – List of primers for GST-3C-Kap95.
Supplementary Figure 1 – NupX and Kap95 SDS-PAGE.
Supplementary Figure 2 – NupX coating of gold surfaces under different concentrations.
Supplementary Figure 3 – SPR measurements of protein- and MUTEG-functionalized chips.
Supplementary Figure 4 – Passivation of NupX-covered Au-surfaces using MUTEG.
Supplementary Figure 5 – Kap95 binding to passivated gold QCM-D chip.
Supplementary Figure 6 – Kap95 dissociation from NupX by 0.2 M NaOH.
Supplementary Figure 7 – Kap95 vs BSA binding to different NupX-coated gold surfaces.
Supplementary Figure 8 – Testing the stability of NupX coatings against milliQ and 50% pure ethanol.
Supplementary Figure 9 – Dissipation-to-Frequency ratio change upon QCM-D coating.
Supplementary Figure 10 – Dissipation-to-frequency ratio of a NupX layer upon increasing Kap95 concentration.
Supplementary Figure 11 – Coarse-grained simulations of cargo adsorption in a NupX brush with a 5.7 nm grafting distance.
Supplementary Figure 12 – Model Kap95 particle.
Supplementary Figure 13 – Conductance decrease upon NupX-coating of solid-state nanopores.
Supplementary Figure 14 – Current Power Spectral Density before and after NupX-coating.
Supplementary Figure 15 – Selectivity measurements through different pore sizes (30 nm, 35 nm and 60 nm).
Supplementary Figure 16 – Event rate of Kap95 translocation through NupX-coated pores vs Kap95 concentration.
Supplementary Figure 17 – Density distributions of NupX for different grafting densities.
Supplementary Figure 18 – Density distributions of NupX in the access region.
Supplementary Figure 19 – Density distribution of NupX in a 30 nm pore in the presence of Kap95 molecules.
Supplementary Figure 20 – Density distributions of NupX variations in nanopores.
Supplementary References

Supplementary Note 1 – Amino acid sequences of all proteins used in this work (NupX, Nsp1, Nsp1-S, Kap95, BSA).

NupX:

MGHHHHHHFNQGSGLLTTFGKASSTSTQASGLFGNGGLNNQQTGFGNIKTGPNPTS
GLFGQTGNSANSSGFGAANTNAFSSGGLFGSQQNGSVQANFGNGSNNGNSTQGLFG
GPMPQTPSAPFGTQQNMTGNNNGLFGSTGGTVTGGSFGNNASASKTQNGLFGDRAR
NQSGFDNPYNKLLRSENSKSEFIVSIDVKSEMAASIKLTAFFGQSTAKNKALSNIGTLI
DKDLKAKKDGTKSRSNPIKLIPNAPFQSKPDNYTPEQDSKNKDSEEETAESAQLLSVQ
VETPDTNTRDLNMSPHSSNSSSMNQLWC

Nsp1:

MSKHHHHSGHHHTGHHHHSGSHHHTGENLYFQGSNFNTPQQNKTPFSFGTANNNS
NTTNQNSSTGAGAFGTGQSTFGFNNSAPNNTNNANSSITPAFGSNNTGNTAFGNSNP
TSNVFGSNNSTTNTFGSNSAGTSLFGSSSAQQTKSNGTAGGNTFGSSSLFNNSTNSNT
TKPAFGGLNFGGGNNTTPSSTGNANTSNNLFGATANANKPAFSFGATTNDDKKTEP
DKPAFSFNSSVGNKTDAQAPTTGFSFGSQLGGNKTVNEAAKPSLSFGSGSAGANPAG
ASQPEPTTNEPAKPALSFGTATSDNKTTNTTPSFSFGAKSDENKAGATSKPAFSFGAK
PEEKKDDNSSKPAFSFGAKSNEDKQDGTAKPAFSFGAKPAEKNNNETSKPAFSFGAK
SDEKKDGDASKPAFSFGAKPDENKASATSKPAFSFGAKPEEKKDDNSSKPAFSFGAK
SNEDKQDGTAKPAFSFGAKPAEKNNNETSKPAFSFGAKSDEKKDGDASKPAFSFGA
KSDEKKDSDSSKPAFSFGTKSNEKKDSGSSKPAFSFGAKPDEKKNDEVSKPAFSFGA
KANEKKESDESKSAFSFGSKPTGKEEGDGAKAAISFGAKPEEQKSSDTSKPAFTFGAQ
KDNEKKTETSC

Nsp1-S:

MSKHHHHSGHHHTGHHHHSGSHHHTGENLYFQGSNSNTPQQNKTPSSSGTANNNS
NTTNQNSSTGAGASGTGQSTSGSNNSAPNNTNNANSSSTPASGSNNTGNTASGNSNP
TSNSSGSNNSTTNTSGSNSAGTSSSGSSSAQQTKSNGTAGGNTSGSSSSSNNSTNSNTT
KPASGGSNSGGGNNTTPSSTGNANTSNNSSGATANANKPASSSGATTNDDKKTEPD
KPASSSNSSSGNKTDAQAPTTGSSSGSQSGGNKTSNEAAKPSSSSGSGSAGANPAGAS
QPEPTTNEPAKPASSSGTATSDNKTTNTTPSSSSGAKSDENKAGATSKPASSSGAKPE
EKKDDNSSKPASSSGAKSNEDKQDGTAKPASSSGAKPAEKNNNETSKPASSSGAKSD
EKKDGDASKPASSSGAKPDENKASATSKPASSSGAKPEEKKDDNSSKPASSSGAKSN
EDKQDGTAKPASSSGAKPAEKNNNETSKPASSSGAKSDEKKDGDASKPASSSGAKS

DEKKDSDSSKPASSSGTKSNEKKDSGSSKPASSSGAKPDEKKNDESSKPASSSGAKA
NEKKESDESKSASSSGSKPTGKEEGDGAKAASSSGAKPEEQKSSDTSKPASTSGAQK
DNEKKTESTSC

Kap95:

MSTAEFAQLLENSILSPDQNIRLTSETQLKKLSNDNFLQFAGLSSQVLIDENTKLEGRI
LAALTLKNELVSKDSVKTQQFAQRWITQVSPEAKNQIKTNALTALVSIEPRIANAAA
QLIAAIADIELPHGAWPELMKIMVDNTGAEQPENVKRASLLALGYMCESADPQSQA
LVSSSNNILIAIVQGAQSTETSKAVRLAALNALADSLIFIKNNMEREGERNYLMQVVC
EATQAEDIEVQAAAFGCLCKIMSLYYTFMKPYMEQALYALTIATMKSPNDKVASMT
VEFWSTICEEEIDIAYELAQFPQSPLQSYNFALSSIKDVVPNLLNLLTRQNEDPEDDD
WNVSMSAGACLQLFAQNCGNHILEPVLEFVEQNITADNWRNREAAVMAFGSIMDG
PDKVQRTYYVHQALPSILNLMNDQSLQVKETTAWCIGRIADSVAESIDPQQHLPGVV
QACLIGLQDHPKVATNCSWTIINLVEQLAEATPSPIYNFYPALVDGLIGAANRIDNEF
NARASAFSALTTMVEYATDTVAETSASISTFVMDKLGQTMSVDENQLTLEDAQSLQ
ELQSNILTVLAAVIRKSPSSVEPVADMLMGLFFRLLEKKDSAFIEDDVFYAISALAASL
GKGFEKYLETFSPYLLKALNQVDSPVSITAVGFIADISNSLEEDFRRYSDAMMNVLA
QMISNPNARRELKPAVLSVFGDIASNIGADFIPYLNDIMALCVAAQNTKPENGTLEAL
DYQIKVLEAVLDAYVGIVAGLHDKPEALFPYVGTIFQFIAQVAEDPQLYSEDATSRA
AVGLIGDIAAMFPDGSIKQFYGQDWVIDYIKRTRSGQLFSQATKDTARWAREQQKR
QLSL

BSA:

MKWVTFISLLLLFSSAYSRGVFRRDTHKSEIAHRFKDLGEEHFKGLVLIAFSQYLQQC
PFDEHVKLVNELTEFAKTCVADESHAGCEKSLHTLFGDELCKVASLRETYGDMADC
CEKQEPERNECFLSHKDDSPDLPKLKPDPNTLCDEFKADEKKFWGKYLYEIARRHPY
FYAPELLYYANKYNGVFQECCQAEDKGACLLPKIETMREKVLASSARQRLRCASIQ
KFGERALKAWSVARLSQKFPKAEFVEVTKLVTDLTKVHKECCHGDLLECADDRAD
LAKYICDNQDTISSKLKECCDKPLLEKSHCIAEVEKDAIPENLPPLTADFAEDKDVCK
NYQEAKDAFLGSFLYEYSRRHPEYAVSVLLRLAKEYEATLEECCAKDDPHACYSTV
FDKLKHLVDEPQNLIKQNCDQFEKLGEYGFQNALIVRYTRKVPQVSTPTLVEVSRSL
GKVGTRCCTKPESERMPCTEDYLSLILNRLCVLHEKTPVSEKVTKCCTESLVNRRPCF
SALTPDETYVPKAFDEKLFTFHADICTLPDTEKQIKKQTALVELLKHKPKATEEQLKT
VMENFVAFVDKCCAADDKEACFAVEGPKLVVSTQTALA

Supplementary Table 1 – Overview of measured Stokes diameters for the protein used by DLS.

Protein	Average Stokes diameter (nm)	Standard deviation (nm)
NupX	7.4	±2.2
Nsp1	14.9	±3.9
Nsp1-S	13.5	±3.1
Kap95	9.2	±1.2
BSA	8.5	±3.3

Hydrodynamic diameter measured with Dynamic Light Scattering (DLS) of the proteins used. Data were all acquired in 150 mM KCl, 10 mM Tris, 1mM EDTA, at pH 7.5, room temperature.

Supplementary Table 2 – Sequences of 25 NupX variants.

Variant number	Sequence
1	HHHHHHTGGGNTPNGNFGGGSAGQANAFGLFGTANGSGQQSGFGQNSNTGTNSNGLFGQNGNFVANASFGTQSGNSPSGSGLFGPKSIMPQPQSFGNAVSSNSTSTGLFGTTTLNQNTTLFGTGNNTGTSPKGLFGMAANNQQGNNFGSGTSLQSQAKGLFGDGLMRKFKKPKQLPGLSNAMLNEHEPSNTLLAAQSPSFISSIKNNNSSKRKNSGEDIIQDNGRTDPNCVFSSINDRYANDDTATATMSPISSAQTYKDDNPAQEKSTSQKTDSKQESKLSFESEKRKLVAEAWLNLNKSPSIFDVLTEV
2	HHHHHHQTAGGQNPNAFGSGQNSLNNPKGLFGSNTNGNQTSTFGPAGTQSSSGSGLFGGNGNQTMQLGFGSGSAQGNSTGGLFGNSSQQVGTNAFGVSTNAGITNQGLFGGQFKNNGTAPFGTKLSSANATNGLFGTASGQPMNTSFGSNGSFNNPTTGLFGDSTLLWITSSLPSDKTPKLSKYRNEAVKDDSPTIVLNNKIVNELKNDGNKNFDQTAKSLGIRLRDLPNRNEQNESNCAVMIDQLFDDPSQSMKAFFSPSQETHAETKSFKELAIKTDSSSGSNASPKSKRSSIAQAGNSYAEKNPQMTE
3	HHHHHHNSGSPNNNQQFGASQTQNAKTTGLFGQTKGATTQSNFGPGNPTIQSNNGLFGGNAMGNSNSTFGSGQNTSLNNSGLFGTGANGSNNQAFGGSGLSSAFTQGLFGNASGVSTGGKFGPTSQLNTAPNGLFGQMTTPGSSGNFGFSANGTVQGGGLFGQSDGRSSKSLNADSYLLNSSHLDNNSDKRNKFPIAFKTPFIKNSQTSSSALAEMFASNKDDPFEQINAQKVIKEIEKLALNMDCNKIQLAPVTSTIPQDSGSDSPSEMTDLKPTSKSKGYWLPGDQSEENVKRNELSKATTRTRNNAVE
4	HHHHHHNVSGVGPSTNFGQGGGTNNTNGGLFGGFNNSSPLSSFGQNPATTSNASGLFGTNNAGTAGGAFGQQKMGQNNQQGLFGSGNNNNSKASFGTNQTGITPSAGLFGSNNGGNTKLQFGGPLQFTTQGQGLFGSSNSMNTTTTFGSASGPSASQAGLFGLLNMFSANTFLTSHDQQIKKPEMDNNGSVETSLNDSRKSRKANPCDIISQTSRSNNIDILNQSSGPQNAKEAIVRNSFPESPDDEKTLLAYKSFNKSEESPSRKSKDTTLTQLSKENWADGMAAGISKVYQKSLNPLKKPASFTEDVDA
5	HHHHHHSTGTTSKTNTFGQQSSLGNALTGLFGSSNPSSAMSSFGAAGNSGGATIGLFGSAGNTNNGQQFGTNPQAANPGMGLFGTTSQNPSKNTFGSQGPSNNGNNGLFGTFSTTTANQNFGLGSGVQNNPVGLFGGNSTSFGQQAFGGNNGQQNKGGGLFGVPNNDESISQPQEENFKDLEGFLSRSKSLLANLEVATIKTEKKSQPVAAKYNSSIKPANPKDNGQISTALPMKFCGPLDSSKSDISSIDMTNDDRTSHWSRENSNTDSTIKALFKLSNVLSQNRAQPKSKDYEQAFLMAKDTNSTNGRE
6	HHHHHHGSAASGSSGNFGGNSAPFSSTQGLFGSSGGIVTNTKFGTNGNQATGTNGLFGFQGQSNQGNKFGNQGMAAATKNGLFGTQTPPGTSNNFGNQSNTPNNGSGLFGTTLGNTTSAAFGAPMLNQSTSGGLFGQSNPQSNQGSFGTNVQGNNSLGGLFGKSQPSIWENSQMDRSPDNSNYEDLMGSPNFYPLKNSADIQLPEKKRKSLTSLKQAIESGFEDSLSDEFRPTDKMKTKNCEFNDKDTKINVAAKIEPASNFKNNLIQIAEGSDVSRNDRAASTTSLSTLTVLHSGQSSPATNNKKASLVQ
7	HHHHHHGSGSPGSGNSFGGQPQGQNGGPGLFGQAQNTSQQAMFGATANMTNTASGLFGGGSNNSSGNTFGTKANNGNVSPGLFGSNSTTNSLNSFGKQLTPNQSLNGLFGTIVTSGNNQTFGNTAGNTPSSQGLFGASSGNGNFGAFGTTTGKQAFNQGLFGDNNNSEIDSFQYFNPETKDVRMKNEEAKFPEPKDLLINPSWGRPKSTLSQSRKFTSNDISTCNEADSKSLSNLSEKQAMVLGRPKLKSNKLSSATEDGMGILAAVEDKSAQDYTSAIPKSQSRFSHNDIKSLKTNNQTAQIPVSALDTN
8	HHHHHHQATVNFNQNAFGAAQAGSMAGNGLFGGGMQSSSNKAFGTINGGGSQNAGLFGTTSGSPNNSTFGTSQSQKGGNTGLFGFSPSQTQGSGFGNGSSTVSNPNGLFGNNGLPTNLQGFGNTSTTNQAGTGLFGTSSPQQPAGNFGNNGTKNSLNTGLFGGDNSDQDINHLISNSKLDSLAESNTLFMKQEEKAKYWKSNPDETLAEKTSNSDTPNINLSLSPTSSNPARAQAQSSMRVILASTTSFKVLTGLKNSENKFKFQPLKQGKMNSINADRCKQSPDSDDPEYKSVRNSETIIVKDARAEPFG

9	HHHHHHGGTASQNNSAFGSGSTTSAQNNGLFGTKSNTGQFPNFGSVGNNTPGSNGLFGSSATGTQQSN<br>FGTNGTNKMPSSGLFGSGAMTLTFNSFGNQVTAGNNGAGLFGNQLTQTNPSQFGGPSANNSNGTGLFG<br>NGLSPQAIKQFGGQGGGQTASNGLFGAQKPKNKAQNGQATDKIPIAAPLFDIWKNLKKLRNSVLSEEK<br>STFKAEPNNQSKKNCLAVPQNDDHSSTKRNEVDTPSALTRISRMSGGNMEKLFLRTNPEIYDESNSSS<br>AESTSKNSDKDIFQNSDYTSQIDATSLSEFLMDLGSVPS
10	HHHHHHFQTKNNGNNSFGTSQTGQGSATGLFGNNANSSPASTFGNSGLATTNNGGLFGGNGNSNNTLG<br>FGSANSQQSQGMGLFGSGTSNPGQATFGNGQSFSAGTNGLFGPQVPGGAKNNFGITTATSQPQPGLFG<br>SSTNTVQTQMFGLAGNSGNSKGGLFGNRDGTIDEAGDSTQKAPSLIELGSELLTESPAATIESLNREE<br>KRTNPKSMLSSYFNEKSKTKLDISNEFNNTKNVSTNSDVPAAAPRPCSKSSLFTNIMQFDKSKDRLFS<br>KNVKLAKPAQDVMQNDQIDDKSKWIHQYLQANPSSSSGN
11	HHHHHHTSGSSNGVNPFGQTNFQPNNVGGLFGGTGGSAPNKNFGAANSNNSTSNGLFGMGNSNQQGSP<br>FGTAAQNSSGTTGLFGGNSNGTPSLSFGTSNSSGTGLNGLFGNPSGNTAQGAFGQQNMSGFGQTGLFG<br>QGTANLQNQIFGSATQTTTKKAGLFGQNNELLSAHDKNKSPKNMAAESANRSFKSLEVQATIIKSNRY<br>GNNSKSILLRFEKEPIMQNPPKTNDPCVTVSLSSDNQLTSSSAEDPASGEFNRTDIWLTESLDKYGGD<br>VATNQAQASSSSIMLLKQDEFIRKDKPTSDDKNSFKPTK
12	HHHHHHAQGQGKKAQSFGLSTTSNSSNNGLFGQNNQTGNQTNFGTGQGQSTANSGLFGSGGNSNTQNG<br>FGAGFPGSPMSVGLFGSNQNGNANGTFGGTSSLNLIPTGLFGNSGNANSMTTFGSTPQVFQATSGLFG<br>NGAGTPPSGTFGGQNSATKNNAGLFGEMNTSVTIEQIDAKKNSDNSFEIESYQSTVRSTWNLKSLQDD<br>NSNSNSPLDGDPIPIRNSQSSYSEDNTDSLKMKTAMKEGELDSERSSGAVDFSNNFIDSNKPICTKKK<br>SQTFTKVGAKQAPKNLAAQHAPLAFKSPKRLENRLLLPA
13	HHHHHHSGGNNGGTSGFGLSVQSQGASPGLFGVQPKTTNGNQFGQNQSQANTGSGLFGPGSSASNNLS<br>FGGNPTAANANAGLFGTGANNSSSIMFGAMTLSNPNNQGLFGKKGNSGTSSNFGQGFTGNNSTQGLFG<br>PTNTQANGFNFGTTGQTQTGTSGLFGCGISNNIYRDESPQSISFSSESSHADENKKNPPSIAKMSDNK<br>SIIVALDSVMVEPTPKATNKDKVFSQSLSQYTKINDSDLAALNEFGQLQKNSELPNSQRLDESSLRPS<br>RNELSRFDKGAQMGKKKDDNNATKWTALLKTTANTFEPT
14	HHHHHHGQQTPSLTKNFGTGSTGTASGAGLFGTNNSTTSVTTFGNGNALSQNNTGLFGGSSQGSPGPN<br>FGNANNNNTGGIGLFGGTTNGMNGFTFGSQASQQSFSNGLFGNSGASSSNASFGPQPQGNQAALGLFG<br>NKSNVNQNPMFGTQGGGKTQASGLFGTSPLNNFQPFQMNQVNNIRSDVLLKQPVWPKSSLSSCQTIDK<br>SRSQNMIAELNDLLAKFDQSIIKEFKSNMSANKTSAENASVRKTDDTSASLEGEYEDLTLDSRYGIGN<br>SDFNKTPPNATDPTNSKDKKLSEIHSAAAKPEERGKKSS
15	HHHHHHLNNSSGNGTNFGSSQSGANNQNGLFGQTNSSGLPQTFGQGTSSTNNTNGLFGGGGQMASFTG<br>FGASAQGSATNVGLFGPQTQQPKNTSFGAGNKGNPGNNGLFGSGTAQGTAFAFGSQANGSSPTNGLFG<br>QVGNTGMNLTFGSSSKTTINNPGLFGLELSFKNKANISTLMWKDKALLKPNASTSQFEKIPDITDSDK<br>FTEAAVKPDDERPKENISSDARLDSSQESHIAKLKSNRLNANLSTGRSKNGVPQATNIQEITQSKSDD<br>YSTPFKVQNNVKLATMSSNPSCSGQNNYRGFLPSMEEDS
16	HHHHHHSMAMFSQTTPFGQGTTQNNPNIGLFGGAALTGASNSFGGQAQSGGTNGGLFGSTNPSQSSKS<br>FGNQSGSGSTNTGLFGQSQQNSNGAAFGNNNNKSGNTAGLFGAGNVGGSNSPFGNTNKGAQVTTGLFG<br>QQTNNSGSTNFGPGPTNGLLTFGLFGPESTSLVSKKDISTIDLANKANNQHDPDNFNPSVKVDKVQPN<br>GDETQPQDLSMELSMPRQKGANQKNASCQERSWKAFSSFKLKNANDTIFSILNKKPLSSLYETKKLSG<br>TRAMTGKDFAESAPTSNDRSSTANLSNIDESIIRELEYS
17	HHHHHHTSNTQGQAGNFGGTPAGSNNSTGLFGITGQNQSGNQFGQGKMNTPQGPGLFGQNSGGSTNNN<br>FGNATSNTVFNSGLFGQNAASQGGNQFGTQNMTTKFANGLFGTNLNVLSPSSFGNGGSSSGNKLGLFG<br>SPSGNSGGASFGAAATSPTQTTGLFGEEQLTSDLNCVPTSDQNNSDIRFTTKQETLSNAGALMPKQSS<br>SDDWNAENKAKYLEIDTEKEQFPINKTPSMDATSNRTVLKERSSFNPRKKASNSFSKDYSFSKNIAKP<br>VISDISGRELKSHSSNDKLQALANINPPAQMDVKLGGSL

18	HHHHHHQGQNQGPSSTFGSSGNQAGVTLGLFGNPSNMNVNQFFGANSTSGAPNGGLFGNTAMNAQTGG<br>FGTGNTSQSTPAGLFGKQSPNGKNATFGITSGGNQSNNGLFGNANGQQKNPFFGLGTSTASSGSGLFG<br>TGNSNTGNTSFGNALSTQSQTGGLFGNLNRNTQINEKSQRASLSSKAISDATSQENTQVNCLNNYNES<br>KSDQNHPVEKKDSAWQKSGTSKTSSESSQKAERSIFDVISFLPLTRDVGSLAKKTTPDFDGENMKFSD<br>ELGKSFRSKANPAPLIAPKNLSDITYDMNAMLPDKLEPI
19	HHHHHHTTASFNNGGTFGSSNPNTSNKGGLFGTSGQNSTSSTFGNQAAQIGGGNGLFGMVQANSNQPP<br>FGAQKNNTQNSTGLFGSQFSGSPLASFGGLTSNTNASTGLFGNATMVNSSSTFGAGQGGNQQQGGLFG<br>QPNLTAGNNGFGPGKTTSGNGNGLFGIQANRSISPNSDQNEHKVKWESSTLDGKTAFDADQSAISAND<br>SNKNIGNKSLPQDGLSCSAAMNPLTSRESSTKLLFSNNKLIIRYERSLFSDRLTENPDSEKDSTMKKN<br>DQKFSKGTVVYQVFPTLPQKEAANPELESKPASITMDNK
20	HHHHHHPPSQNNQKQTFGNQNSTANNSSGLFGANNGTSQAANFGVNQNTGTQTSGLFGSSTSQAQSGG<br>FGTTPAGQTNNTGLFGGNTNANNPGSFGTSKGNPIGQAGLFGGQGSFASKPAFGVMSGNGNSGGGLFG<br>SSQSTFGNLLFGNGTLSTMTNGGLFGQSDKNSNSISDFNCTIDDKAITKVLAKALQNKEDVFRPNSSK<br>KAVSPKSKSETINDDLVRTSPDGYTESLSILNSSSAMPRKGTTLLTAQFKSQLYEFPDENSKDRKPKS<br>MDWSNERGKNGENLISAFQLANQISMPAATSENLQPHNE
21	HHHHHHVQGNGGQKTQFGTSSNNAGPNQGLFGQAKNTNSQGQFGAGPAGPTNSNGLFGTNGNSNAGGQ<br>FGKNMSFSNFGTGLFGSSNQAQLPASFGSTNVSNPTQPGLFGTNSIATAMSGFGQGGSSGTNANGLFG<br>NGSTSNTNLTFGTTSLQTSGNGGLFGKQARPSSADPQIKSSMDSSQVDPLQNKPEKLSHDEANLKPLS<br>PDEEKFITYINTSRQNELKSSKELYIMNDGWGNRNKKFDSSTSKIFSNFTTEANRLNELDGDLAPKSN<br>DKMPRTSVAKSNSFQVSAETSGNCKTTQIDNAASILVLA
22	HHHHHHPTSTTATSNTFGNTSNNNNGNGGLFGTNQKTSQNTSFGGGILTAGTSSGLFGFFNGNTTNKA<br>FGPPGGNAQGASGLFGSPAPQGKSSGFGSGNTNMNSQTGLFGMQSGQTGQLSFGQSPANANNQNGLFG<br>SGGANVSSALFGTNVSGQGNQQGLFGTGSRAFKRPINSSCDETPSNAIKNQLKSKDGKNDDRDNPDVQ<br>TEANESSMAQSAELDTPAKYREDFIFQSELKTANALKTDASLGNSDLINQTSVDSPLLESIQSKKLTT<br>PLSRMNFKLSMKKESSKNHGSPEWVAFSQKYINVNSINP
23	HHHHHHNNTTQPNQNNFGLSQGSTQTSSGLFGSQQMNNGTNPFGGQNGTGSSAGGLFGQGGNGSGTST<br>FGGAGKNNTGFPGLFGPKAAGSNLPSFGGNSLTQNATGGLFGTAISTPVQSTFGAMNNSTGASNGLFG<br>KNTGNSTQVNFGFSSAQQNNSAGLFGLEKSSENSMTDKFSAKSSISKVAQEAVPVLGARPSDFAKFNP<br>TYGPSKLQLNTDQEAIRKNSKQQNTMSENDTFKTRVNELNDQQSDWACPNRHDSKNEETKALILAPGS<br>NASPTNYSILLIDLKFSEPKNISSMLSDKSNIGDTSRKD
24	HHHHHHASGLASSSNGFGTSQTTGTSQPGLFGANSKGGQAPAFGGAGSPNGNQTGLFGQSMQSTAGTA<br>FGNTNNMTSKGQGLFGGTNQSANQNSFGPVGNSNGNGPGLFGNGNQGTKQNSFGQLQATNTLNGGLFG<br>TVNNTSPSNNFGITSFNSFGTSGLFGRNNISLSKYLRKLNCEPAKGFSRKEAATTWPQSKSAKINDES<br>SQASQTMITVNFSTSSVAETKKELSPNFSQSSLFDDIKHSYADTEDSSADGTIVPPPVDLENLNQSGS<br>MSAKNKGDLDKQKPKNKNNEFEARLLQDMINNSDITRPL
25	HHHHHHAQGNTSQNAGFGQPTSTGTTNFGLFGTNSTAQQNNNFGGQQAAPSGNNGLFGLSSNSTLTKT<br>FGTNNIQNPSASGLFGNKSASSAVSGFGMGLNVQSGGAGLFGQNPNSNGGTNFGSPNGKQGTSNGLFG<br>NGTPMGANSSFGGTGTFTSQGQGLFGSLISPENTERSEIPTKVWSKVGSPKPIFNYLQTSLSTLINQN<br>KSYQSLQSSGRFNKNSKEPALELLRDFDGKEMENLTNKKDDSKDDNRNKTKSTCMQASPFASLASHFD<br>NTKGDDPIEDASVSEVQAIAMSPRTKAAKLSNIANQDSN

Supplementary Table 3 – Simulation parameters used for MD-simulations.

System description	System size (no. of beads)	Number of simulations	Forcefield	Timestep (ps)	Simulation time (steps)	Temperature (K)
NupX protein	311	1	1-BPA	0.02	5.00E+08	300
NupX variants	311	25	1-BPA	0.02	5.00E+08	300
NupX brush, 5.7 nm spacing	12060	1	1-BPA	0.02	1.75E+08	300
NupX brush, PMF, Kap	12233	46	1-BPA	0.02	5.00E+07	300
NupX brush, PMF, Kap equilibration	12233	46	1-BPA	0.02	5.00E+06	300
NupX brush, PMF, inert particle	12223	47	1-BPA	0.02	5.00E+07	300
NupX brush, PMF, inert particle equilibration	12223	47	1-BPA	0.02	5.00E+06	300
NupX brush, 4 nm spacing	11991	1	1-BPA	0.02	1.75E+08	300
NupX brush, PMF, Kap	12164	51	1-BPA	0.02	5.00E+07	300
NupX brush, PMF, Kap equilibration	12164	51	1-BPA	0.02	5.00E+06	300
NupX brush, PMF, inert particle	12154	51	1-BPA	0.02	5.00E+07	300
NupX brush, PMF, inert particle equilibration	12154	51	1-BPA	0.02	5.00E+06	300
NupX nanopore, 15 nm	11407	1	1-BPA	0.015	2.00E+08	300
NupX nanopore, 15 nm, equilibration	11407	1	1-BPA	0.015	5.00E+06	270
NupX nanopore, 20 nm	15551	1	1-BPA	0.015	2.00E+08	300
NupX nanopore, 20 nm, equilibration	15551	1	1-BPA	0.015	5.00E+06	270
NupX nanopore, 25 nm	19694	1	1-BPA	0.015	2.00E+08	300
NupX nanopore, 25 nm, equilibration	19694	1	1-BPA	0.015	5.00E+06	270
NupX nanopore, 30 nm	23834	1	1-BPA	0.015	4.00E+08	300
NupX nanopore, 30 nm, equilibration	23834	1	1-BPA	0.015	5.00E+06	270
NupX nanopore, 35 nm	27977	1	1-BPA	0.015	2.00E+08	300
NupX nanopore, 35 nm, equilibration	27977	1	1-BPA	0.015	5.00E+06	270
NupX nanopore, 40 nm	32121	1	1-BPA	0.015	2.00E+08	300
NupX nanopore, 40 nm, equilibration	32121	1	1-BPA	0.015	5.00E+06	270
NupX nanopore, 45 nm	36260	1	1-BPA	0.015	2.00E+08	300
NupX nanopore, 45 nm, equilibration	36260	1	1-BPA	0.015	5.00E+06	270
NupX nanopore, 50 nm	39160	1	1-BPA	0.015	2.00E+08	300
NupX nanopore, 50 nm, equilibration	39160	1	1-BPA	0.015	5.00E+06	270
NupX nanopore, 60 nm	47443	1	1-BPA	0.015	2.00E+08	300
NupX nanopore, 60 nm, equilibration	47443	1	1-BPA	0.015	5.00E+06	270
NupX nanopore, 70nm	55726	1	1-BPA	0.015	2.00E+08	300
NupX nanopore, 70 nm, equilibration	55726	1	1-BPA	0.015	5.00E+06	270
NupX variants in nanopores, 30nm	23834	25	1-BPA	0.015	2.00E+08	300
NupX variants in nanopores, 30nm (equilibration)	23834	25	1-BPA	0.015	5.00E+06	270
30nm NupX nanopore with 10 Kap95p particles and cylindrical occlusion	38574	1	1-BPA	0.015	3.33E+08	300
30nm NupX nanopore with 10 inert particles and cylindrical occlusion	38474	1	1-BPA	0.015	3.33E+08	300

Supplementary Table 4 – List of primers for GST-3C-Kap95.

Name	Sequence
ed7	5’-TTCCAGGGGCCCGCATCTGTTGGATCCATGTCCACC-3’
ed8	5’-CAGAACTTCCAGATCCGATTTTGGAGGATGGTC-3’

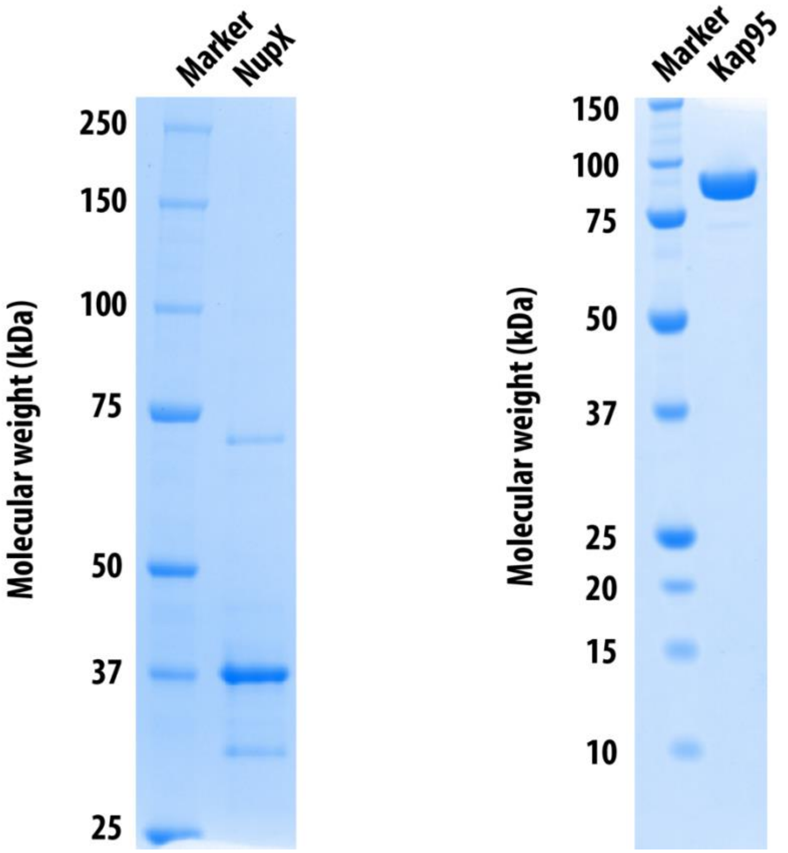

Supplementary Figure 1 – NupX and Kap95 SDS-PAGE.

SDS-PAGE gel shows the band for the 32.5 kDa NupX molecule (left, thick bend running at ~37 kDa) and the 95kDa Kap95 molecule (right) after purification. This experiment was reproduced more than three times yielding similar results.

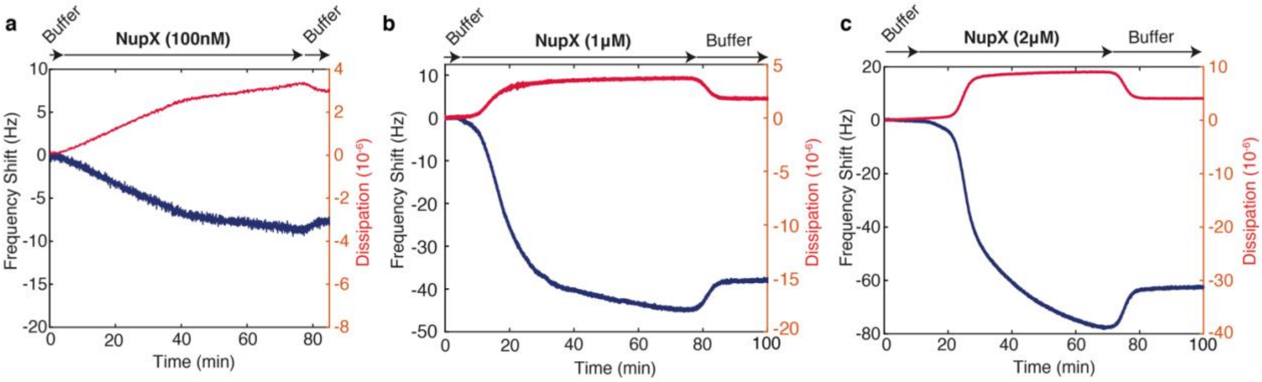

Supplementary Figure 2 – NupX coating of gold surfaces under different concentrations.

Real-time monitoring of NupX-coating of gold-coated quartz chips under concentrations of 100 nM (**a**), 1 µM (**b**), and 2 µM (**c**) at constant flow-rate (20 µL/min), using QCM-D. The slight increase in frequency at the end of the incubation represents the washing step, which induces a subsequent release of nonspecifically bound NupX molecules. The NupX solution included also 1 mM of TCEP in order to reduce the cysteines, which was present during the coating step.

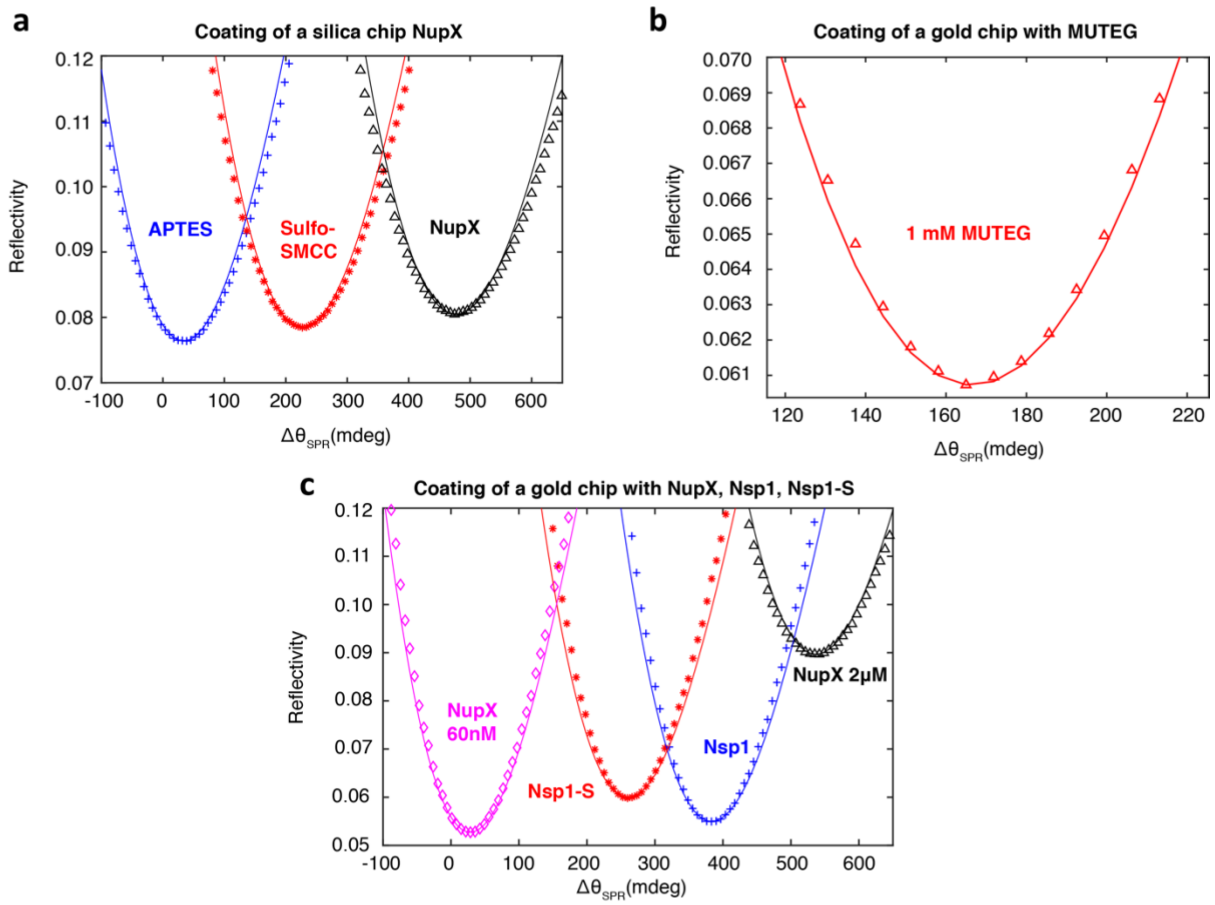

Supplementary Figure 3 – SPR measurements of protein- and MUTEG-functionalized chips.

Angular reflectivity spectra of SPR measurements for dried samples in air. **a** Data for APTES (blue crosses), APTES + Sulfo-SMCC (red asterisks), and APTES + Sulfo-SMCC + NupX (black triangles) on silicondioxide-coated sensors. **b** Data for MUTEG (380 Da) on gold sensors grafted using 1 mM concentration. **c** Data for 1 µM Nsp1 (blue crosses), 1 µM Nsp1-S (red asterisks), 2 µM (black triangles) and 60 nM NupX (pink diamonds) on gold sensors. $\Delta\theta_{SPR}$ denotes the angular shift in millidegrees of the resonance angle. Solid lines show Fresnel model fits (which are offset in the y-direction to match the measurement reflectivity minima), which are used to determine the thickness of each adlayer.

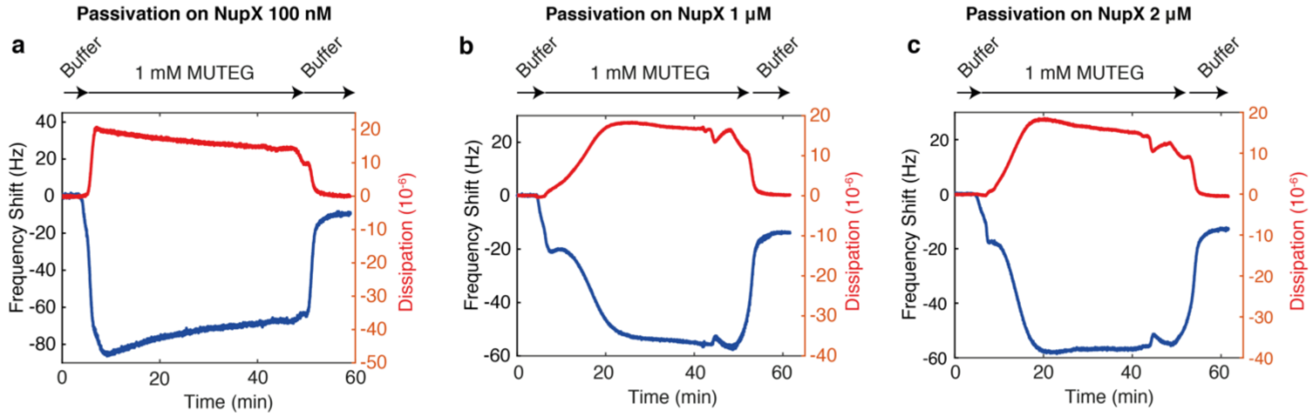

Supplementary Figure 4 – Passivation of NupX-covered Au-surfaces using MUTEG.

Real-time monitoring of 1 mM MUTEG binding to gold chips that were pre-functionalized with NupX at NupX concentrations of 100 nM (**a**), 1 µM (**b**), and 2 µM (**c**) at constant flow-rate (20 µL/min), using QCM-D. Note that final dissipation shifts were all close to zero, while (negative) frequency shifts were found in the range of ~10-14 Hz, indicating the formation of a thin monolayer. The MUTEG solution included also 10 mM of TCEP in order to reduce the thiols, which was present during the coating step (same for **Supplementary** Fig. 5a).

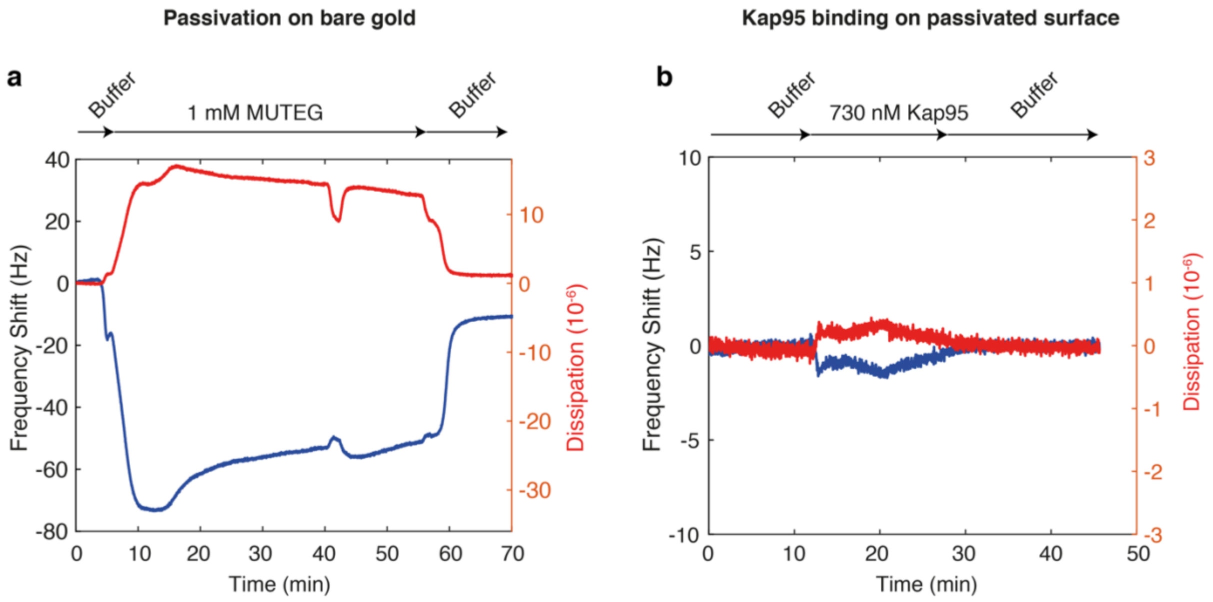

Supplementary Figure 5 – Kap95 binding to passivated gold QCM-D chip.

Real-time monitoring of Kap95 interacting with a MUTEG-passivated gold surface. **a**, Frequency shift over time upon flushing of 1 mM of 1-mercapto-11-undecyltetra(ethyleneglycol) (MUTEG) onto a gold surface. From an independent measurement using SPR (Supplementary Fig. 3b) where the same incubation time and concentration were used, the MUTEG coating of a bare gold surface yielded a dense monolayer with a 0.59 ± 0.01 nm average grafting distance. **b**, Frequency shift over time upon flushing of 730 nM of Kap95 to a MUTEG-passivated gold layer. Only minor interactions (< 2 Hz) were detected.

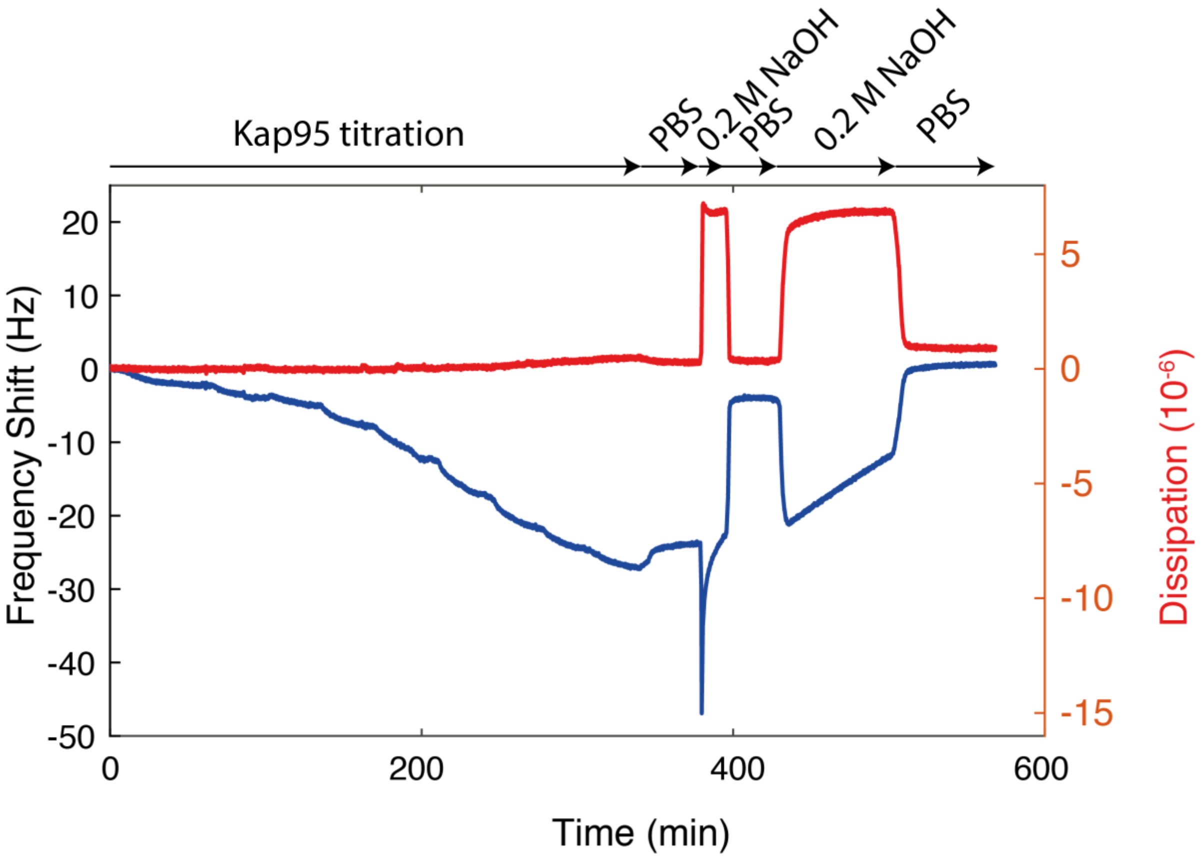

Supplementary Figure 6 – Kap95 dissociation from NupX by 0.2 M NaOH.

Real-time monitoring of Kap95 interacting with a NupX-coated surface with subsequent dissociation upon flushing of 0.2 M NaOH.

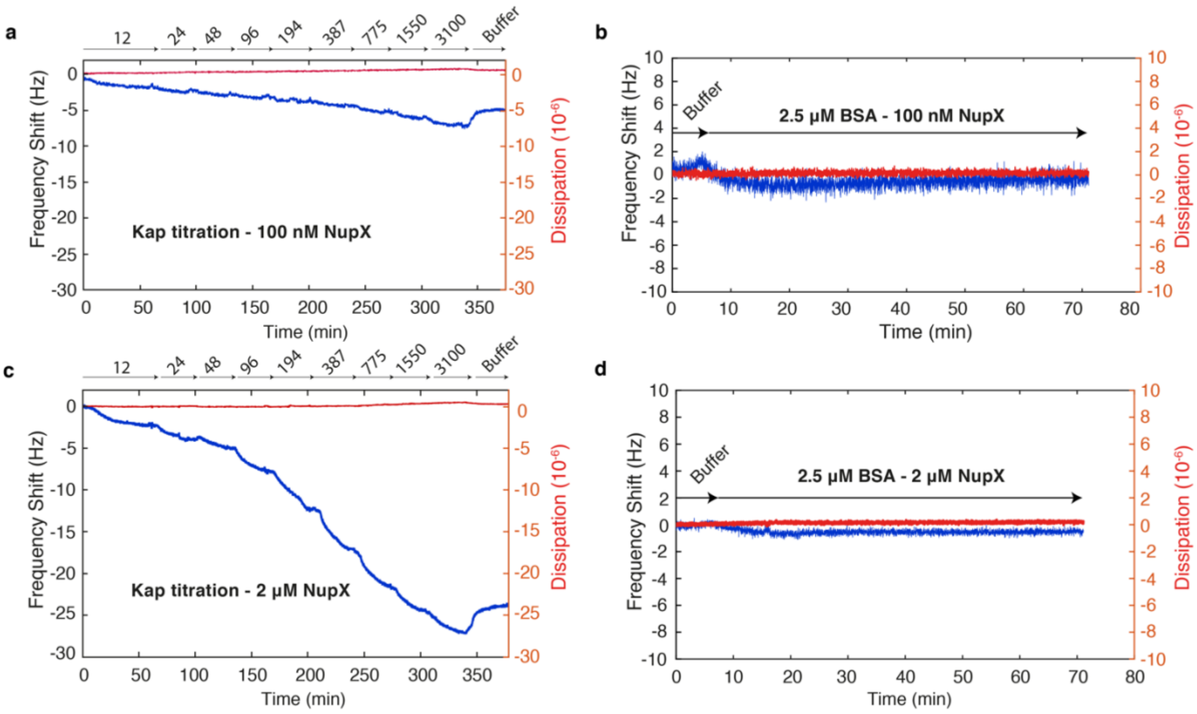

Supplementary Figure 7 – Kap95 vs BSA binding to different NupX-coated gold surfaces.

Real-time monitoring of ~10-3000 nM Kap95 and 2500 nM BSA binding to gold chips functionalized with NupX at a concentration of 100 nM (**a**-**b**, respectively) and 2 µM (**c**-**d**, respectively), at constant flow-rate (20 µL/min), using QCM-D.

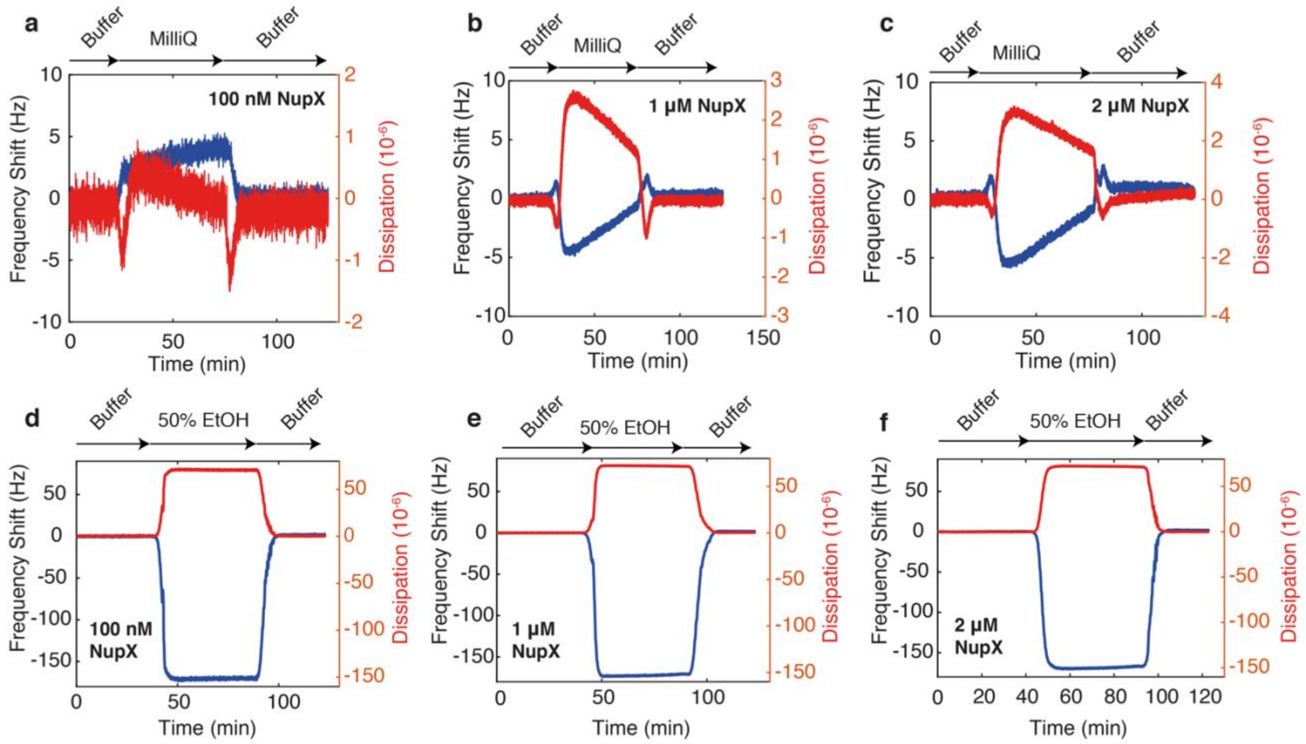

Supplementary Figure 8 – Testing the stability of NupX coatings against milliQ and 50% pure ethanol.

Real-time monitoring of milliQ and 50% pure ethanol rinsing of gold surfaces that were pre-coated with NupX and subsequently passivated with 1 mM MUTEG, at different NupX concentrations of 100 nM (**a**,**d**, respectively), 1 µM (**b**,**e**, respectively), and 2 µM (**c**,**f**, respectively), at constant flow-rate (20 µL/min), using QCM-D. Note that final frequency and dissipation shifts are all close to zero, indicating that our protein and MUTEG layers are stably bound to the gold surface.

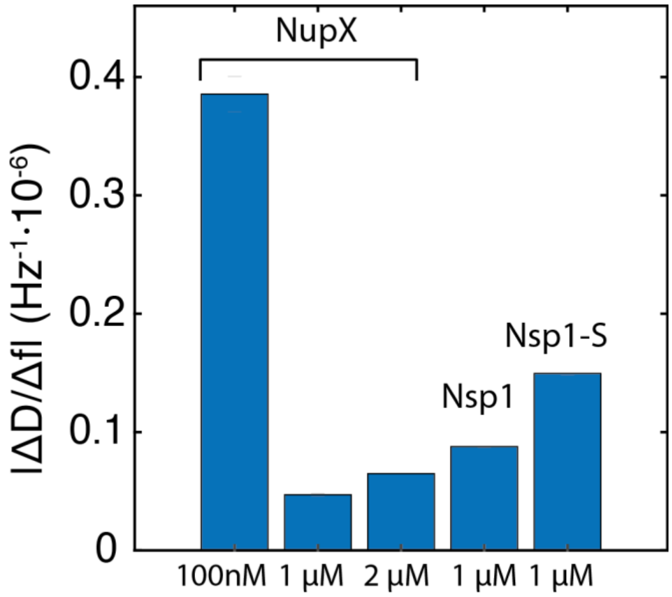

Supplementary Figure 9 – Dissipation-to-Frequency ratio change upon QCM-D coating.

Dissipation-to-frequency ratio $\Delta D_5/(\Delta f_5/5)$ for NupX (with concentrations as in Supplementary Figure 2), Nsp1, Nsp1-S coating of a gold-coated quartz chip. When comparing the 3 proteins (NupX, Nsp1, and Nsp1-S) for the same incubation concentration of 1 µM, NupX produced the lowest dissipation-to-frequency ratio, consistent with the higher hydrophobic character of GLFG-type Nups (e.g. Nup98[1]), and hence of NupX, as compared to FXFG-Nups. Note that these $\Delta D_5/(\Delta f_5/5)$ values are for the FG-Nup films only, i.e. prior MUTEG passivation.

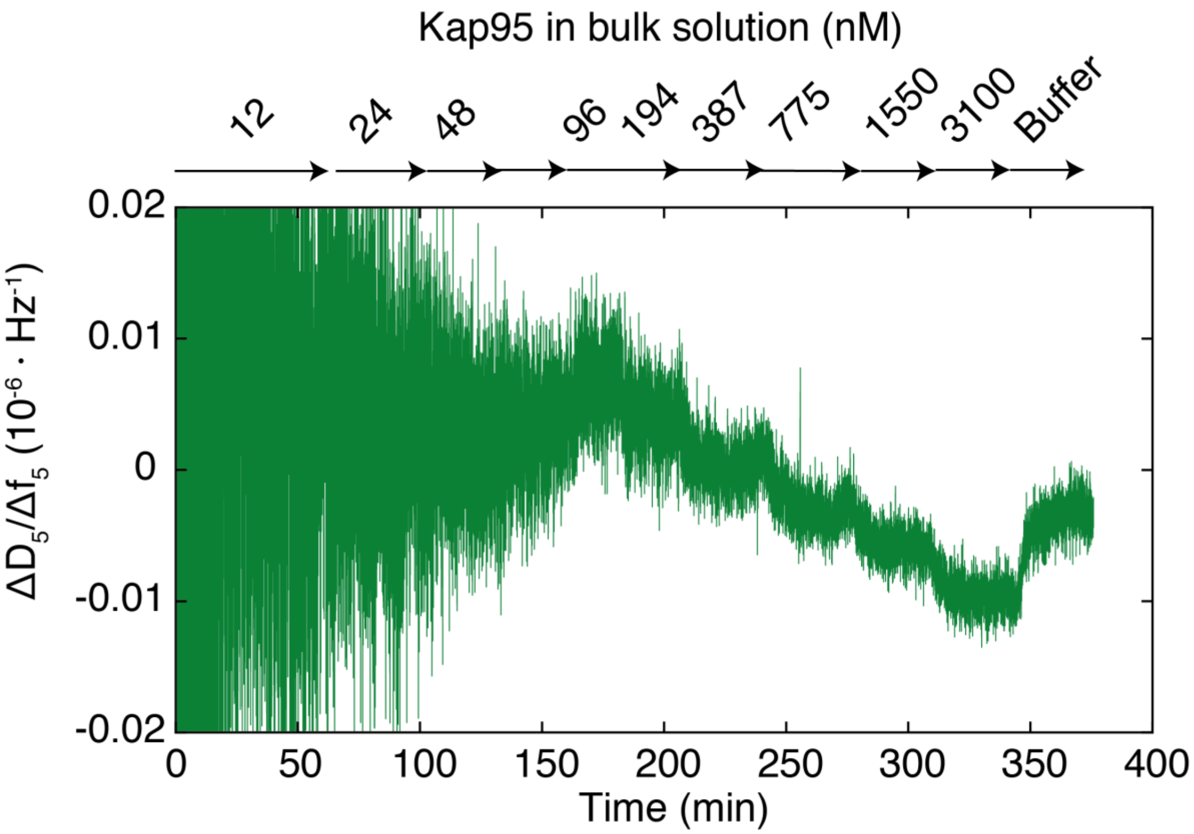

Supplementary Figure 10 – Dissipation-to-frequency ratio of a NupX layer upon increasing Kap95 concentration.

Dissipation-to-frequency ratio $\Delta D_5/(\Delta f_5/5)$ for Kap95 absorption to a NupX-coated surface for increasing concentrations (from 12 nM to 3100 nM).

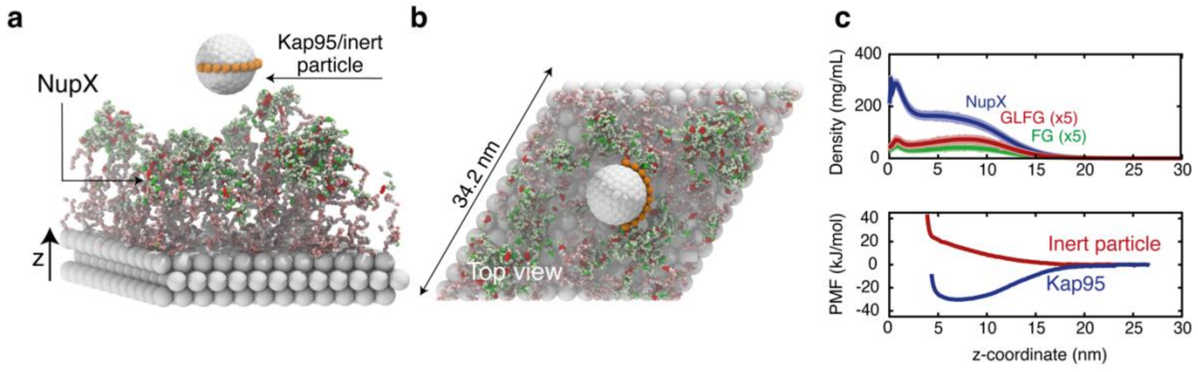

Supplementary Figure 11 – Coarse-grained simulations of cargo adsorption in a NupX brush with a 5.7 nm grafting distance.

NupX brush system, simulated for a grafting distance of 5.7 nm. **a,b** Snapshots of the simulations (see also: Figure 2h). **c**, Top panel: Time and laterally averaged protein density distributions for the NupX brushes and for the two different types of FG motifs present inside the NupX brushes. The density profiles of the GLFG and FG motifs within the NupX brush are multiplied by 5 for clarity of display. Dark central lines and light shades indicate the mean and standard deviation in density profiles, respectively. These measures were obtained by averaging over the density profiles of trajectory windows 50 ns in length (N=60). A high-density region (up to a maximum of 300 mg/mL) forms near the attachment sites of the NupX to the surface ($z = 0$ to 2 nm). Further away from the scaffold, the protein density remains at a constant value of ~ 170 mg/mL up to a distance of ~8 nm, after which it decays. FG and GLFG motifs predominantly localize near the transitioning point (8 nm). Bottom panel: Free-energy profiles (PMF-curves) of the center of mass of the model Kap95 and inert particle along the z-coordinate, where $z = 0$ coincides with the substrate. The PMF-curves originate from a z-value where the distance between the center of mass of either particle and the substrate approaches one half of the former's diameter. The difference in sign between the PMF-curves of both particles indicates a strong preferential absorption of the model Kap95 to NupX brushes and a repulsive interaction of the inert particle. Compared to the brush with a lower grafting distance (see Figs. 2h,i in the main text), the repulsion and adsorption are less strong, which is due to the decrease in the density of the NupX brush and FG/GLFG-motifs, respectively (see **c** top panel).

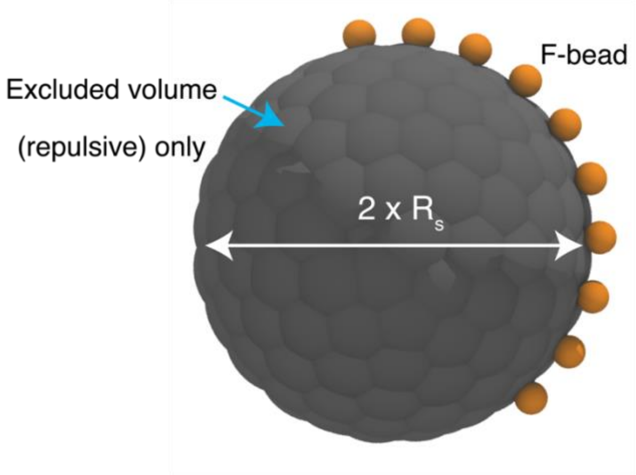

Supplementary Figure 12 – Model Kap95 particle.

The computational model of the Kap95 particle used in this work, rendered using VMD[2]. The Kap95 (and the inert particle) consists of sterically repulsive beads (i.e., only repulsive excluded volume interactions with its surroundings, here shown in dark grey) arranged in a geodesic shell. In the case of the Kap95 particle a strip of 10 hydrophobic binding sites (brown-orange) is placed on the surface, and the net charge (-43e) is distributed equally over all the surface beads. Binding sites are modeled as Phenylalanine beads and are spaced 1.3 nm apart on an arc. Phe-beads are the most hydrophobic particle type in our 1-BPA model. The diameter of Kap95 is equal to two times the Stokes radius (see Supplementary Table 1).

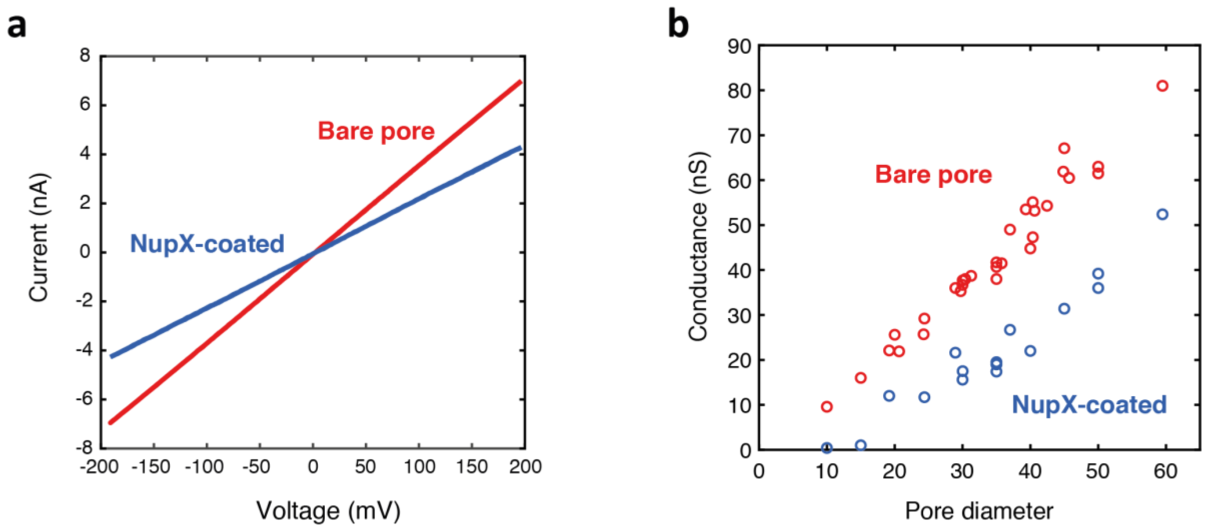

Supplementary Figure 13 – Conductance decrease upon NupX-coating of solid-state nanopores.

NupX-coating of solid-state nanopores. **a,** I-V characteristics for a bare (red) and NupX-coated (blue) 30 nm pore. **b,** Ionic conductance of differently sized nanopores over a range of 10-60 nm is plotted vs diameter for bare (red) and coated (blue) pores.

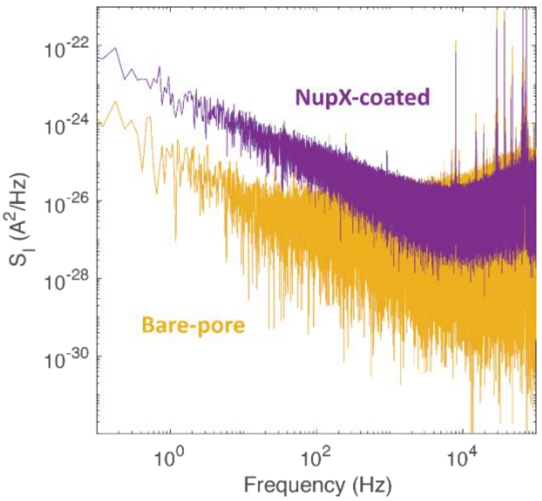

Supplementary Figure 14 – Current Power Spectral Density before and after NupX-coating.

Power Spectral Density of the ionic current noise before (yellow curve) and after (purple) NupX-coating for a 30 nm pore.

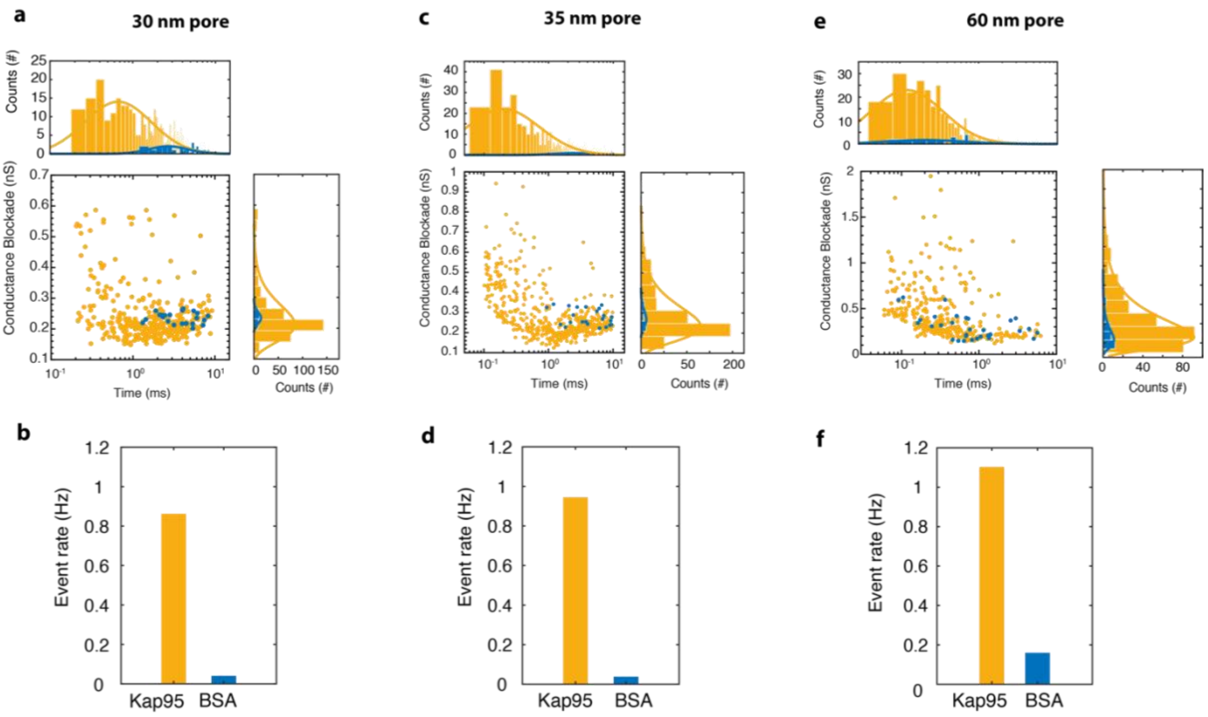

Supplementary Figure 15 – Selectivity measurements through different pore sizes (30 nm, 35 nm and 60 nm).

Selectivity measurements on NupX-coated pores of different sizes. Scatter plots show event distributions for translocations of 2.8 µM BSA and 450 nM Kap95 under 100 mV applied bias, in 150 mM KCl, pH 7.4 buffer. **a-b**, Scatter plot and bar plot showing (**a**) conductance blockades (0.24±0.07 nS for Kap95, 0.24±0.02 nS for BSA; errors in s.d.) *vs* dwell times (2.4±0.4 ms for Kap95, 4.2±1.2 ms for BSA; errors in s.e.m.) (**b**) and event rates (0.9 Hz for Kap95, 0.04 Hz for BSA) for translocations of Kap95 (N=369) and BSA (N=22) through a NupX-coated 30 nm pore. **c-d**, Scatter plot and bar plot showing (**c**) conductance blockades (0.3± 0.1 nS for Kap95, 0.28±0.04 nS for BSA; errors in s.d.) *vs* dwell times (2.5±0.2 ms for Kap95, 5.2±0.9 ms for BSA; errors in s.e.m.) and (**d**) event rates (0.95 Hz for Kap95, 0.04 Hz for BSA) for translocations of Kap95 (N=482) and BSA (N=23) through a NupX-coated 35 nm. **e-f**, Scatter plot and bar plot showing (**e**) conductance blockades (0.45± 0.03 nS for Kap95, 0.30±0.02 nS for BSA; errors in s.d.) *vs* dwell times (0.65±0.05 ms for Kap95, 1.6±1.3 ms for BSA; errors in s.e.m.) and (**f**) event rates (1.1 Hz for Kap95, 0.04 Hz for BSA) for translocations of Kap95 (N=314) and BSA (N=32) through a NupX-coated 60 nm pore.

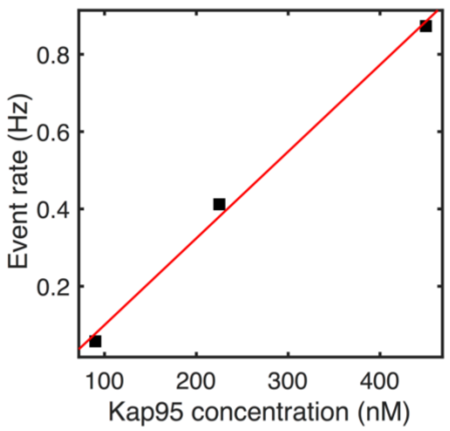

Supplementary Figure 16 – Event rate of Kap95 translocation through NupX-coated pores vs Kap95 concentration.

Event rate of translocation of Kap95 molecules through a NupX-coated pore, at increasing concentrations from 90 nM, 225 nM, to 450 nM. The translocation frequency increases linearly with concentration, as expected.

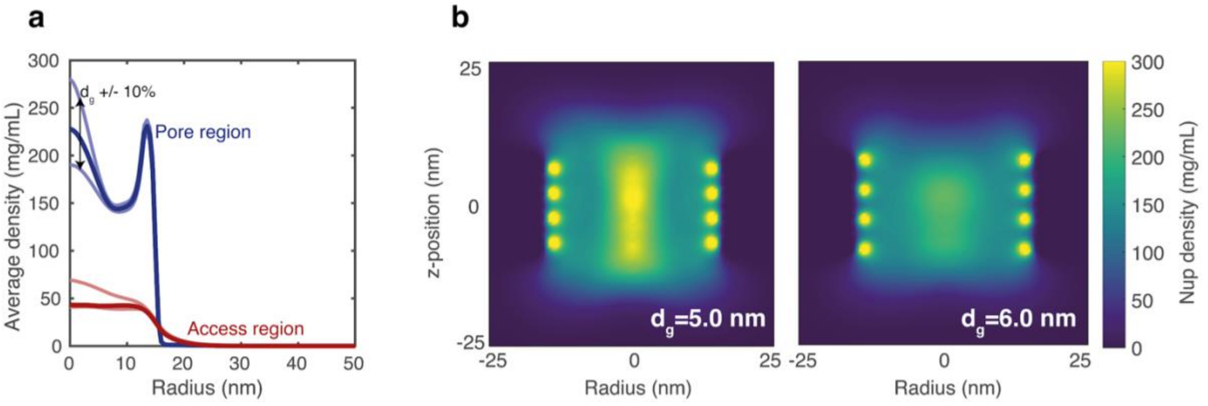

Supplementary Figure 17 – Density distributions of NupX for different grafting densities.

Sensitivity of the average pore and access region protein densities against a change in grafting distance d_g. **a,** Upon increase resp. decrease of the grafting distance with approx. 10% (6.0/5.0 nm as compared to 5.5 nm used for SiN in the main text), we observe a corresponding decrease (-17%) and increase (+22%) in the maximal pore region density (light blue and dark blue curves, resp.). The maximal access region density (red) is only sensitive to a decrease in grafting distance, where a 10% decrease in grafting distance yields a large density increase of +60% due to NupX proteins being expelled from the pore region (light red). **b**, Axi-radial density distributions of 30 nm NupX-lined nanopores upon an approximate 10% decrease (left panel) or increase (right panel) in grafting distance. The high-density region towards the central axis of the nanopore increases in density and extends over a larger range in the z-direction when the grafting distance is decreased, whereas the opposite occurs for an increase in grafting distance. The overall morphology of the NupX-meshwork in the nanopore remains consistent under an increase of approximately 10% in grafting distance.

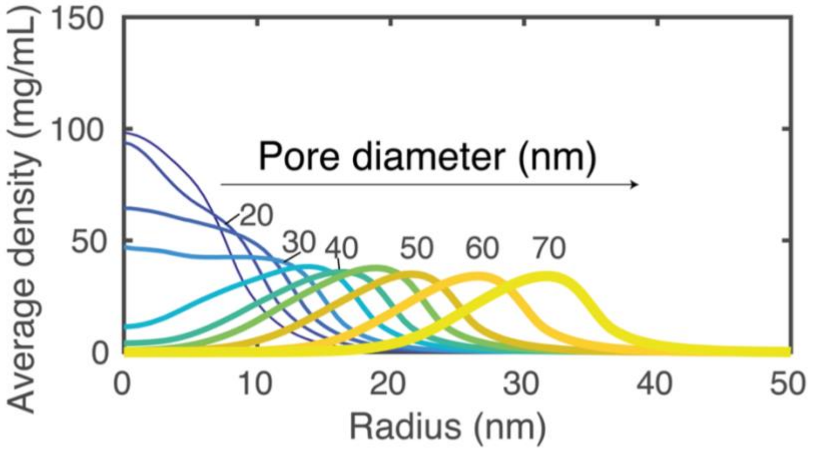

Supplementary Figure 18 – Density distributions of NupX in the access region.

Access region densities for NupX-lined nanopores with diameters ranging from 15 nm to 70 nm. Lighter colors and increasing line thicknesses indicate larger diameter pores. A preferred localization of NupX proteins towards the central axis of the pore region occurs for a pore diameter of 30 nm and smaller.

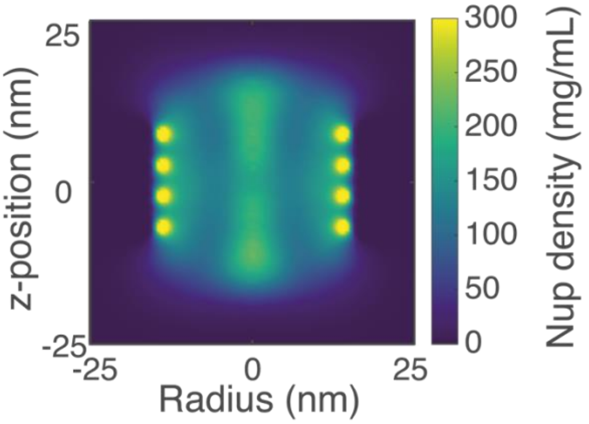

Supplementary Figure 19 – Density distribution of NupX in a 30 nm pore in the presence of Kap95 molecules.

Axi-radial density map of the protein density distribution in a 30 nm NupX-lined nanopore that interacts with Kap95 particles. The density distribution shifts towards the interface between the pore and access regions, rather than focus centrally in the pore, which is the case when no Kap95 particles are present (as shown in Figure 4b).

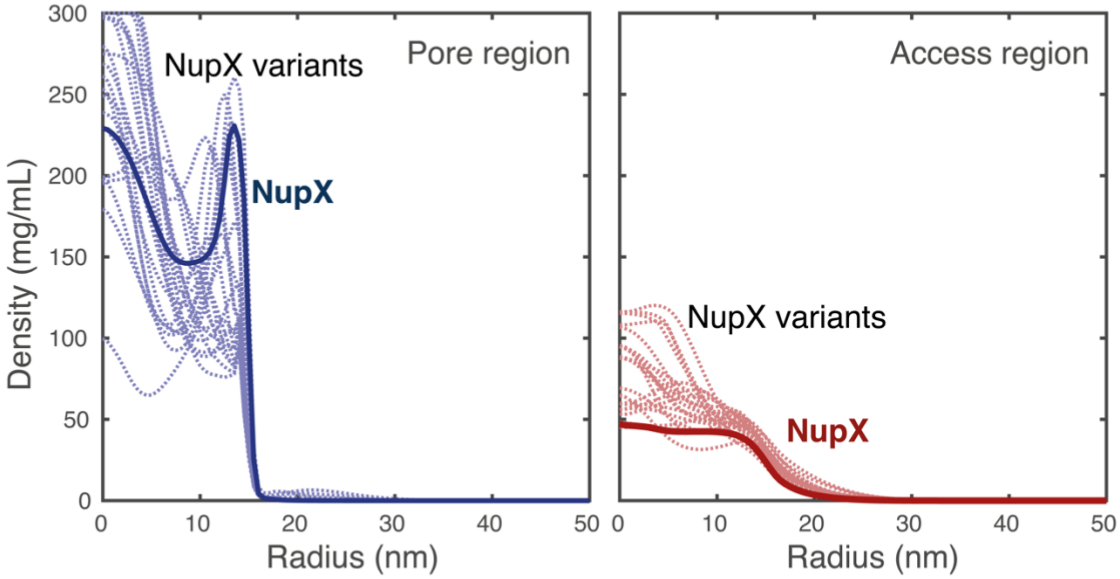

Supplementary Figure 20 – Density distributions of NupX variations in nanopores.

Density distributions in the pore (blue dotted lines, left) and access (red dotted lines, right) regions for 25 NupX variants from Supplementary Table 2. NupX (bold lines) is shown for reference.

Supplementary References

1. Eisele, N. B., Labokha, A. A., Frey, S., Görlich, D. & Richter, R. P. Cohesiveness tunes assembly and morphology of FG nucleoporin domain meshworks - Implications for nuclear pore permeability. *Biophys. J.* **105**, 1860–1870 (2013).
2. Humphrey, W., Dalke, A. & Schulten, K. VMD: Visual molecular dynamics. *J. Mol. Graph.* **14**, 33–38 (1996).
