# Supplementary material for: A designer FG-Nup that reconstitutes the selective transport barrier of the nuclear pore complex
Source: Nat Commun. 2021 Mar 31;12:2010. doi: 10.1038/s41467-021-22293-y (PMC8012357; doi:10.1038/s41467-021-22293-y)
Supplement: Supplementary file 3 — Reporting Summary [file 41467_2021_22293_MOESM3_ESM.pdf]

nature research

Corresponding author(s): C. Dekker
P. R. Onck

Last updated by author(s): Feb 3, 2021

Reporting Summary

Nature Research wishes to improve the reproducibility of the work that we publish. This form provides structure for consistency and transparency in reporting. For further information on Nature Research policies, see our Editorial Policies and the Editorial Policy Checklist.

Statistics

For all statistical analyses, confirm that the following items are present in the figure legend, table legend, main text, or Methods section.

n/a	Confirmed	
☐	☒	The exact sample size (*n*) for each experimental group/condition, given as a discrete number and unit of measurement
☒	☐	A statement on whether measurements were taken from distinct samples or whether the same sample was measured repeatedly
☒	☐	The statistical test(s) used AND whether they are one- or two-sided<br>*Only common tests should be described solely by name; describe more complex techniques in the Methods section.*
☒	☐	A description of all covariates tested
☒	☐	A description of any assumptions or corrections, such as tests of normality and adjustment for multiple comparisons
☐	☒	A full description of the statistical parameters including central tendency (e.g. means) or other basic estimates (e.g. regression coefficient) AND variation (e.g. standard deviation) or associated estimates of uncertainty (e.g. confidence intervals)
☒	☐	For null hypothesis testing, the test statistic (e.g. *F*, *t*, *r*) with confidence intervals, effect sizes, degrees of freedom and *P* value noted<br>*Give P values as exact values whenever suitable.*
☒	☐	For Bayesian analysis, information on the choice of priors and Markov chain Monte Carlo settings
☒	☐	For hierarchical and complex designs, identification of the appropriate level for tests and full reporting of outcomes
☒	☐	Estimates of effect sizes (e.g. Cohen's *d*, Pearson's *r*), indicating how they were calculated

Our web collection on statistics for biologists contains articles on many of the points above.

Software and code

Policy information about availability of computer code

Data collection	Coarse-grained MD simulations were carried out using the open-source GROMACS package, version 2016.3 on a parallelized high performance computer cluster. Disorder profiles and assessments of secondary structure were obtained using the web-portals of PONDR (http://www.pondr.com), DISOPRED3 (http://bioinf.cs.ucl.ac.uk/psipred/) and Phyre2 (http://www.sbg.bio.ic.ac.uk/~phyre2).<br>QCM-D data were collected with Q-soft, software provided by the company Biolin Scientific together with the QMC-D device. Nanopore data were collected with Clampex v9.2.<br>SPR data was collected from raw data text-files produced by Bionavis MP-SPR Navi 220A instrument.
Data analysis	Data from MD simulations were analyzed using built-in tools from the open-source GROMACS 2016.3 package, the freely available HYDRO++ software version 10 or in-house MATLAB and Python codes. Visualizations of our simulations were obtained using the freely available VMD software, version 1.9.3.<br>GLFG-Nup sequences were analysed using a custom-written R script.<br>QCM-D data were analysed and fitted using Qtools, software provided by the company Biolin Scientific. Raw data from QCM-D were smoothened and plotted in MATLAB. Nanopore data were analyzed with custom-written MATLAB script, which is described in published literature.<br>SPR analysis was performed using custom-written MATLAB code previously described in published literature.

For manuscripts utilizing custom algorithms or software that are central to the research but not yet described in published literature, software must be made available to editors and reviewers. We strongly encourage code deposition in a community repository (e.g. GitHub). See the Nature Research guidelines for submitting code & software for further information.

Data

Policy information about availability of data

All manuscripts must include a data availability statement. This statement should provide the following information, where applicable:

- Accession codes, unique identifiers, or web links for publicly available datasets
- A list of figures that have associated raw data
- A description of any restrictions on data availability

Source data for Figs. 1d-h, 2a-g,i, 3c-g, 4b-d,f-h, and 5a,b,e are provided with the paper in Supplementary Table S5.
Other data that support the findings of this study are available from the corresponding authors upon reasonable request.

Field-specific reporting

Please select the one below that is the best fit for your research. If you are not sure, read the appropriate sections before making your selection.

[x] Life sciences [ ] Behavioural & social sciences [ ] Ecological, evolutionary & environmental sciences

For a reference copy of the document with all sections, see nature.com/documents/nr-reporting-summary-flat.pdf

Life sciences study design

All studies must disclose on these points even when the disclosure is negative.

Sample size	QCM-D data were repeated more than 3 times and we consistently obtained similar results. Nanopore data points in scatter-plots were between 300-1000, which is a statistically relevant sample size for typical nanopore measurements. Such sample sizes were suitable given the estimated error was low as compared to the mean. Bare pore and NupX-coated pore selectivity measurements were performed more than three times each for pores of ~30 nm and the given BSA and Kap95 concentration, which yielded similar results in terms of event rates. SPR data was repeated 3 times for all measurements except for MUTEG measurements, where each concentration was measured 1 time.
Data exclusions	We excluded data for clogged nanopores or broken nanopore chips as they were not relevant for the study, since the correct reconstitution of the biomimetic system failed and was not possible to acquire the data. We excluded data for QCM-D experiments where baseline resonance frequency was dramatically unstable or drifting, since the acquired data would have otherwise been convoluted with unwanted noise signal. We excluded one SPR measurement on MUTEG concentration measurements due to being a significant outlier.
Replication	Data could be readily reproduced once similar conditions for the experiment were successfully met. For QCM-D experiments, similar grafting density for the FG-Nup layer and Kap and BSA concentrations led to similar behaviors and have been reproduced for independent experiments more than 3 times. For nanopores, upon ensuring proper NupX coating and wetting of the chip that allowed to measure the ionic conductance through the pore, independent experiments led to similar behaviors and have been reproduced more than 3 times. For SPR experiments, repeated measurements where performed to ensure static conditions inside the measurement cell and the reported data were reproduced at least 3 times.
Randomization	It was not relevant for our QCM-D, SPR or nanopore experiments because of how the experiments were designed. The use of control experiments ensured that the acquired data reflected the interpreted behavior.
Blinding	It was not relevant for our QCM-D, SPR or nanopore experiments because of how the experiments were designed. The use of control experiments ensured that the acquired data reflected the interpreted behavior.

Reporting for specific materials, systems and methods

We require information from authors about some types of materials, experimental systems and methods used in many studies. Here, indicate whether each material, system or method listed is relevant to your study. If you are not sure if a list item applies to your research, read the appropriate section before selecting a response.

Materials & experimental systems

n/a	Involved in the study	
[x]	[ ]	Antibodies
[x]	[ ]	Eukaryotic cell lines
[x]	[ ]	Palaeontology and archaeology
[x]	[ ]	Animals and other organisms
[x]	[ ]	Human research participants
[x]	[ ]	Clinical data
[x]	[ ]	Dual use research of concern

Methods

n/a	Involved in the study	
[x]	[ ]	ChIP-seq
[x]	[ ]	Flow cytometry
[x]	[ ]	MRI-based neuroimaging
